# An Interesting Case of Nonlupus Full-House Nephropathy

**DOI:** 10.1155/2021/9043003

**Published:** 2021-12-28

**Authors:** Satyanand Sathi, Alok Sharma, Anil Kumar Garg, Virendra Singh Saini, Manoj Kumar Singh, Devinder Vohra, Arvind Trivedi

**Affiliations:** ^1^Department of Medicine, S.M.M.H. Government Medical College, Saharanpur, Uttar Pradesh, India; ^2^Department of Renal Pathology and Electron Microscopy, Dr Lal Path-lab Limited, New Delhi, India

## Abstract

Full-house immunofluorescence and endothelial tubuloreticular inclusions are known as characteristic features of lupus nephritis. However, both features are not pathognomonic for lupus nephritis. A kidney biopsy specimen showing full-house immunofluorescence pattern in the absence of autoantibodies and classical clinical features of Systemic Lupus Erythematosus (SLE) is now considered as nonlupus full-house nephropathy (FHN). Nonlupus FHN may be idiopathic or due to other disease processes known as secondary nonlupus FHN. Here, we report the case of a 36-year-old female who presented with nephrotic proteinuria with bland urine sediment. Additional analyses revealed normal serum antinuclear antibody (ANA), normal anti-double-stranded DNA (anti-dsDNA) antibodies, and normal serum C3 and C4 levels. A renal biopsy showed a normal-appearing glomerulus without any proliferation or capillary wall thickening and widespread glomerular immune deposits (full-house effect; IgA, IgG, IgM, C3, and C1Q) on direct immunofluorescence. Renal electron microscopy showed diffuse effacement of visceral epithelial cell foot processes and mesangial electron dense deposits. The patient was diagnosed as nonlupus FHN. There is a controversial role of steroids and other immunosuppressive drugs in the treatment of nonlupus FHN patients, but our case patient responded favourably to steroid therapy. The term nonlupus FHN can be used as an umbrella term for patients who do not satisfy the clinical and serological criteria of SLE.

## 1. Introduction

The American College of Rheumatology (ACR) classifications require at least four clinical and serological criteria for the diagnosis of Systemic Lupus Erythematosus (SLE). However, according to SLICC (Systemic Lupus International Collaborating Clinics) classification, antinuclear antibody (ANA) and/or anti-double-stranded DNA (anti-dsDNA) antibody positivity in the presence of kidney biopsy consistent with lupus nephritis is a criterion sufficient to diagnose SLE [1]. A variety of kidney lesions can be caused by SLE. In a kidney biopsy specimen, if all five major immunofluorescent stains (IgA, IgG, IgM, C3, and C1Q) are positive, then it is considered as full-house immunofluorescence [2]. Full-house immunofluorescence and endothelial tubuloreticular inclusions are known as characteristic features of lupus nephritis [3]. However, both features are not pathognomonic for lupus nephritis [3]. Tubuloreticular inclusions (TRIs) have also been associated with human immunodeficiency virus-associated nephropathy (HIVAN) and rarely with C1Q nephropathy [4]. Wen and Chen, first of all, systematically illustrated nonlupus full-house nephropathy in a group of twenty-four patients [3]. A kidney biopsy specimen showing full-house immunofluorescence pattern in the absence of autoantibodies and classical clinical features of SLE is now considered as nonlupus full-house nephropathy (FHN) [5]. Nonlupus FHN may be idiopathic or due to other disease processes (secondary) [5]. According to some anecdotal reports, full-house nephropathy may be the first presentation of SLE without any clinical and serological evidence [6, 7]. Formerly, these cases were misclassified as seronegative lupus nephritis when the serum autoantibodies were negative for SLE [8]. Over years of follow-up, seroconversion has been reported from negative to positive lupus serology only in a minority of these patients [5, 6, 8]. Nonetheless, the majority of these patients do not develop any clinical and serological evidence of SLE over a period of time [7]. According to the underlying glomerular disease processes, steroids and other immunosuppression may be used, but poor renal outcome was observed in idiopathic nonlupus full-house nephropathy patients [5, 8].

## 2. Case Presentation

A 36-year-old female presented with a 3-year history of on and off swelling fully over the body without any history of sore throat, fever, joint pain, hematuria, rash, oral ulcers, alopecia, breathlessness, seizures, psychosis, chest pain, miscarriage, smoking, alcohol drinking, and NSAID abuse. There was no history of type 2 diabetes mellitus and hypertension.

**Figure 1:**
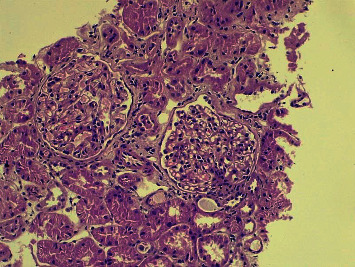
Kidney biopsy specimen on light microscopy showing a normal-appearing glomerulus without any proliferation or capillary wall thickening.

**Figure 2:**
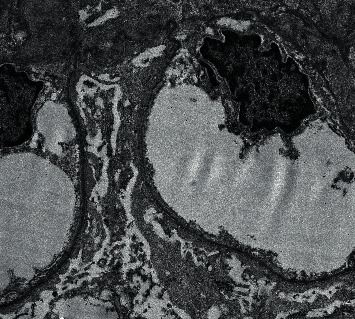
Renal electron microscopy showing diffuse effacement of visceral epithelial cell foot processes and mesangial electron-dense deposits.

The patient's laboratory profile was as follows: hemoglobin: 13.9 g/dL, total leukocyte count: 8,300/mm3, platelet count: 3.9 × 10^5^/mm^3^, urinary albumin: 4+, urinary sugar: 0, urine microscopy: white blood cell count: 4–5/high-power field, red blood cell count: 1–2/high-power field, urinary pH: –6.5, urine culture: sterile, serum albumin: 1.5 g/dL, serum sodium: 136.6 mEq/L, serum potassium: 3.8 mEq/L, serum calcium: 8.5 mg/dL, serum phosphorus: 3.4 mg/dL, random blood sugar: 108 mg/dL, blood urea: 28 mg/dL, serum creatinine: .7 mg/dL, serum antistreptolysin O titer (ASO titer): <100 IU/mL, C3: 149.3 mg/dL (normal range: 90–180), C4: 46.40 mg/dL (normal range: 10–40), serum antinuclear antibody: negative, anti-dsDNA antibodies: 11.17 IU/ml (normal <30 IU/ml), perinuclear antineutrophil cytoplasmic antibody: negative, cytoplasmic antineutrophil cytoplasmic antibody: negative, HIV I and II: negative, HBsAg: negative, and anti-HCV: negative. A 24-hour urinary protein value was 6.8 grams/day. Ultrasonography abdomen showed bilateral normal-size kidneys with normal echogenicity. A renal biopsy showed nonproliferative glomerulopathy (15 glomeruli), with no evidence of segmental sclerosis and tuft sclerosis (Figure 1). Tubular atrophy involved less than 10% of the sampled cortex. Tubules showed focally prominent cytoplasmic vacuolar changes, and the arteries sampled appeared unremarkable. Direct immunofluorescence showed widespread glomerular immune deposits (full-house effect). The following immunostaining pattern was observed: IgA: 2+ mesangial; granular, IgG: 2+ mesangial; granular, IgM: 2+ mesangial; granular, C3: 2+ mesangial; granular, C1Q: 2+ mesangial; granular, kappa light chains: 2+ mesangial; granular, and Lambda light chains: 2+ mesangial; granular. Renal electron microscopy showed diffuse effacement of visceral epithelial cell foot processes and mesangial electron dense deposits (Figure 2). Thus, in view of the full-house pattern of immunofluorescence and in the absence of autoantibodies and classical clinical features of SLE, the diagnosis of nonlupus full-house nephropathy was made. We initiated treatment with prednisone 1 mg/kg/day. After 4 weeks of steroid therapy, 24-hour urine protein was 1.4 grams/day. After 8 weeks of steroid therapy, proteinuria was undetectable in a 24-hour urine sample. Steroid was tapered gradually over a period of 6 months.

## 3. Discussion

Our case patient did not show any clinical and serological evidence of SLE and did not satisfy criteria of the American College of Rheumatology (ACR) classification to make a diagnosis of SLE [9]. The etiopathogenesis of nonlupus FHN is not elucidated and may have similarity to lupus nephritis. There may be polyclonal B-cell activation with the expression of a more striking type of defective clearance of immune complexes and immune complex handling or overloading of abnormal immune complexes [5]. The secondary forms of nonlupus FHN are possibly associated with these mechanisms. Unknown exogenous or endogenous antigens and genetic factors such as deficiency of the erythrocyte C3b receptor and Fcc receptor deficiency may be related to the etiopathogenesis of idiopathic nonlupus FHN [5, 10].

On the basis of light microscopy, the most common cause of nonlupus full-house nephropathy was described as membranous nephropathy followed by IgA nephropathy, infection-related glomerulonephritis, membranoproliferative glomerulonephritis, diffuse proliferative glomerulonephritis, crescentic glomerulonephritis, amyloidosis, and C1Q nephropathy [5, 8]. On light microscopy, a kidney biopsy specimen of our case patient showed nonproliferative glomerulopathy, with no evidence of segmental sclerosis and tuft sclerosis, and there was a full house of glomerular immune deposits on immunofluorescence. The possible differential diagnosis for our case patient would be idiopathic nonlupus FHN or minimal change disease variant of C1Q nephropathy or a rare scenario of turning out to be class 2 lupus nephritis with podocytopathy during long-term follow-up. Rijnink et al. studied 32 nonlupus FHN patients and out of these 32 patients, 20 patients had idiopathic nonlupus FHN and 12 patients had secondary nonlupus FHN [5]. Out of 20 idiopathic nonlupus FHN patients, two patients showed minimal change lesions on light microscopy, and out of 12 secondary nonlupus FHN patients, one patient showed minimal change lesion on light microscopy [5]. During a median follow-up of 20 years, none of the 32 nonlupus FHN patients developed SLE as shown by the study conducted by Rijnink et al. [5]. C1Q nephropathy is usually prevalent in children and young adults [11]. Our case patient was a 36-year-old adult female. C1Q nephropathy is considered as a controversial and poorly understood clinical entity [11]. C1Q nephropathy was found to be the least common cause of secondary nonlupus full-house nephropathy in the study conducted by Wani et al. [8]. Asymptomatic hematuria, subnephrotic to nephrotic proteinuria, hypertension, and renal insufficiency are common presenting features of C1Q nephropathy [11]. There are two histological subtypes of C1Q nephropathy: minimal change disease (MCD)/focal segmental glomerulosclerosis (FSGS) and proliferative glomerulonephritis [11]. Our case patient presented with nephrotic proteinuria without any hematuria, hypertension, and renal insufficiency but on light microscopy showed an MCD-like lesion. On electron microscopy, the presence of amorphous electron dense deposits in the mesangium ± glomerular capillary wall is considered as a consistent finding in all cases of C1Q nephropathy irrespective of their light microscopic subtype [11]. Vizjak et al. reported that on electron microscopy, out of 53 patients of C1Q nephropathy, 48 patients had mesangial or mesangial subendothelial or mesangial subendothelial-subepithelial or mesangial subepithelial deposits [12]. In our case patient, there were no subepithelial, subendothelial, and glomerular capillary wall deposits on electron microscopy. Hardly ever, tubuloreticular cytoplasmic inclusions may be found in glomerular and peritubular capillary endothelial cells in C1Q nephropathy [11].

Some definite characteristics (but not pathognomonic) are usually observed only in lupus nephritis. These include endothelial tubuloreticular inclusions on electron microscopy, a full-house immunofluorescence pattern, and membranous glomerulonephritis with mesangial deposits [3]. On electron microscopy, our case patient did not show any evidence of endothelial tubuloreticular inclusions and membranous nephropathy but showed diffuse effacement of visceral epithelial cell foot processes with mesangial electron dense deposits. Wen and Chen told that, out of 59 patients with nonlupus full-house nephropathy reported in the literature, only seven patients developed serological or clinical evidence of SLE. [3]. Moreover, it has been documented recently that circulating anti-C1Q antibodies are characteristic features of lupus nephritis and these antibodies are absent in C1Q nephropathy patients [13]. This test was not performed in our case patient as we do not have the facility to test for anti-C1Q antibodies.

There is a controversial role of steroids and other immunosuppressive drugs in the treatment of nonlupus FHN patients. Rijnink et al. reported poor renal outcome in idiopathic FHN patients. Ruggiero et al. observed a favourable renal outcome in nonlupus FHN patients who received cytotoxic immunosuppression [14]. High proportion of steroid resistance has been observed in C1Q nephropathy patients [15]. In steroid resistant cases of C1Q nephropathy, a good response has been noticed for cyclophosphamide, azathioprine, cyclosporine, mycophenolate, and tacrolimus when used separately or in combination with steroids [11]. However, our case patient responded favourably to steroid therapy [16].

## 4. Conclusions

The full-house pattern of renal involvement has been described most commonly in SLE, but rarely can occur in other diseases, including an idiopathic variant. The term nonlupus FHN can be used as an umbrella term for patients who do not satisfy the clinical and serological criteria of SLE, and it will avoid the misclassification of patients as seronegative lupus nephritis. Nephrologists should give attention to these patients as a minority of these patients would convert to SLE during long-term follow-up.

## Data Availability

The datasets used and/or analyzed during the current case report are available from the corresponding author upon reasonable request.

## References

[1] Inês L., Silva C., Galindo M. (2015). Classification of systemic lupus erythematosus: systemic Lupus International Collaborating Clinics versus American College of Rheumatology Criteria. a comparative study of 2,055 patients from a real-life, international systemic lupus erythematosus cohort. *Arthritis Care & Research*.

[2] Cameron J. S. (1999). Lupus nephritis. *Journal of the American Society of Nephrology*.

[3] Wen Y.-K., Chen M.-L. (2010). Clinicopathological study of originally non-lupus “full-house” nephropathy. *Renal Failure*.

[4] Elmaghrabi A., Brown E., Khin E., Hassler J., Hendricks A. R., Hendricksa (2017). Tubuloreticular inclusions in the absence of Systemic Lupus Erythematosus and HIV infection: a report of three pediatric cases. *Case Reports in Nephrology and Dialysis*.

[5] Rijnink E. C., Teng Y. K. O., Kraaij T., Wolterbeek R., Bruijn J. A., Bajema I. M. (2017). Idiopathic non-lupus full-house nephropathy is associated with poor renal outcome. *Nephrology Dialysis Transplantation*.

[6] Gianviti A., Barsotti P., Barbera V., Faraggiana T., Rizzoni G. (1999). Delayed onset of systemic lupus erythematosus in patients with “full-house” nephropathy. *Pediatric Nephrology*.

[7] Baskin E., Agras P. I., Menekşe N., Ozdemir H., Cengiz N. (2007). Full-house nephropathy in a patient with negative serology for lupus. *Rheumatology International*.

[8] Wani A. S., Zahir Z., Gupta A., Agrawal V. (2020). Clinicopathological pattern of non-lupus full house nephropathy. *Indian Journal of Nephrology*.

[9] Hochberg M. C. (1997). Updating the American College of Rheumatology revised criteria for the classification of systemic lupus erythematosus. *Arthritis and Rheumatism*.

[10] Zuniga R., Ng S., Peterson M. G. (2001). Low-binding alleles of Fcc receptor types IIA and IIIA are inherited independently and are associated with systemic lupus erythematosus in Hispanic patients. *Arthritis & Rheumatism*.

[11] Devasahayam J., Erode-Singaravelu G., Bhat Z., Oliver T., Chandran A., Zeng X. (2015). C1q nephropathy: the unique underrecognized pathological entity. *Analytical Cellular Pathology*.

[12] Vizjak A., Ferluga D., Rožič M. (2008). Pathology, clinical presentations, and outcomes of C1q Nephropathy. *Journal of the American Society of Nephrology*.

[13] Sharman A., Furness P., Feehally J. (2004). Distinguishing C1q nephropathy from lupus nephritis. *Nephrology Dialysis Transplantation*.

[14] Ruggiero B., Vivarelli M., Gianviti A. (2017). Outcome of childhood-onset full-house nephropathy. *Nephrology Dialysis Transplantation : Official Publication of the European Dialysis and Transplant Association - European Renal Association*.

[15] Gunasekara V. N., Sebire N. J., Tullus K. (2014). C1q nephropathy in children: clinical characteristics and outcome. *Pediatric Nephrology*.

[16] Sathi S., Garg A. K., Singh A. K., Singh M. K., Saini V. S. (2019). Postinfectious glomerulonephritis with crescents in an elderly diabetic patient after acute gastroenteritis: case report. *Case Reports in Nephrology and Dialysis*.

